# Tutor auditory memory for guiding sensorimotor learning in birdsong

**DOI:** 10.3389/fncir.2024.1431119

**Published:** 2024-07-01

**Authors:** Yoko Yazaki-Sugiyama

**Affiliations:** Neuronal Mechanism for Critical Period Unit, OIST Graduate University, Okinawa, Japan

**Keywords:** auditory, song learning, critical period, sensorimotor learning, songbird, template matching

## Abstract

Memory-guided motor shaping is necessary for sensorimotor learning. Vocal learning, such as speech development in human babies and song learning in bird juveniles, begins with the formation of an auditory template by hearing adult voices followed by vocally matching to the memorized template using auditory feedback. In zebra finches, the widely used songbird model system, only males develop individually unique stereotyped songs. The production of normal songs relies on auditory experience of tutor’s songs (commonly their father’s songs) during a critical period in development that consists of orchestrated auditory and sensorimotor phases. “Auditory templates” of tutor songs are thought to form in the brain to guide later vocal learning, while formation of “motor templates” of own song has been suggested to be necessary for the maintenance of stereotyped adult songs. Where these templates are formed in the brain and how they interact with other brain areas to guide song learning, presumably with template-matching error correction, remains to be clarified. Here, we review and discuss studies on auditory and motor templates in the avian brain. We suggest that distinct auditory and motor template systems exist that switch their functions during development.

## Introduction

**FIGURE 1:**
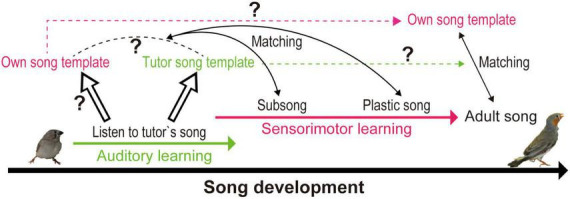
Conceptual diagram of the zebra finch song learning process: template formation and template matching. Juveniles form a tutor auditory template by listening to tutor’s songs, which may facilitate formation of own song motor template. Juveniles develop their own adult songs by matching their vocalization to the template (unclear whether tutor song template or own song template), using auditory feedback. Template matching with auditory feedback is believed to continue until adulthood, as disturbing auditory feedback leads to song degradation.

Sensorimotor learning depends on memory formation, followed by matching a motor pattern to the memorized template. When learning to speak, human babies shape their auditory detection skills based on the sensory environment. Later, they sculpt their vocalization using auditory feedback, which is restricted within the range of acquired auditory perception. Similalrly, songbirds learn to sing first by memorizing a tutor’s songs (TS), commonly their father’s songs, and then by matching their vocalizations to the memorized TS via auditory feedback during the song-learning period (Figure 1). Depending on the bird species, only males or both sexes sing to attract mating partners, to identify their territory, and to facilitate individual recognition. Early behavioral studies in white crowned sparrows (*Zonotrichia leucophrys*), which form memories and start to sing in different seasons, explained beautifully the multistep process of song learning; isolation after hearing and memorizing TS in the spring does not prevent juveniles from developing normal adult songs and learning from TS when they start to sing in the fall. If juveniles are isolated from TS before auditory learning or are deafened in the period between auditory and sensorimotor learning, song learning fails to develop properly. These behavioral studies emphasize the importance of forming auditory memories of TS by listening to the tutor and the necessity of auditory feedback during sensorimotor learning (Konishi, 1965; Marler, 1970).

In zebra finches (*Taeniopygia guttata)*, the widely used songbird model system, only males sing, and females do not. Male zebra finches develop individually unique songs in the largely overlapping auditory and sensorimotor learning phases and retain them throughout their life. Their songs are similar, but never identical, to TS, which is important for identity recognition. Sensorimotor learning ends irrespectively of the level of similarity between own song and TS or song maturity. Thus, the pre-determined time course of song learning based on age is suggested. These observations raise questions on how error signals shape the motor pattern of singing, especially in later phases of song learning, if auditory memories of TS instruct sensorimotor learning and regulate the song-learning time window. Alternatively, do birds construct a “motor template” by hearing TS? The “template” and “template matching” theories have been discussed in songbird research for decades, but only vague and occasionally confusing definitions of song templates (auditory memory of TS or a bird’s own motor pattern) have been provided. In this review, we sought to discuss how and where in the brain song templates are stored to be utilized for song learning.

## “Template matching” theory

In describing the song template system, Peter Marler determined in his early studies that templates serve as filters to detect own species song first, and later for the formation of memories of TS and motor learning guidance; these definitions suggest that templates have multiple functions depending on the developmental time course (Marler, 1970). Marler later described preactive (active) and latent templates. The former normally acts as a filter for preferential learning from own species songs and later guides song learning if birds are not exposed to adult songs, while the latter guides motor learning with respect to the formed memory of adult songs (Marler, 1984; Marler and Nelson, 1992; Marler, 1997). Marler and others, with slight variations, have indicated that template formation requires auditory experiences of conspecific adult songs in addition to innate predispositions. Normally birds learn to sing by hearing TS, suggesting that memories of TS function as templates, while isolated birds use an internal song model as template [reviewed in Mooney (2009)]. While isolated songs feature abnormal acoustic characteristics, such as longer duration and limited variety of syllables with relatively simple features, prolonged isolation (over generations) somewhat normalizes songs, suggesting the presence of innate predispositions (Fehér et al., 2009). Templates may facilitate memory formation of TS (or memories of TS are “templates” as themselves) and for shaping motor learning later. Whether auditory memory of TS and motor templates, namely the preactive and latent templates described in the early work of Peter Marler, are distinct has yet to be clarified.

TS memories are believed to be formed for song learning (tutor templates), as social isolation (absence of tutor template) leads to abnormal song development. However, zebra finches do not develop an exact copy of TS for their own song development. Each individual among siblings intentionally develops own unique songs by coping distinct parts of father’s songs (Tchernichovski and Nottebohm, 1998). Additionally, song learning concludes regardless of the level attained (similarity to TS), but depending on a developmental time course. These notions indicate that template matching with error correction with TS auditory memories alone would not lead to the development of individual songs and suggest that motor templates must be established. In the following sections, we discuss the brain circuits that host specific song templates and aspects of their temporal development.

## Song motor template in the premotor area, HVC, the apex of the song system

**FIGURE 2:**
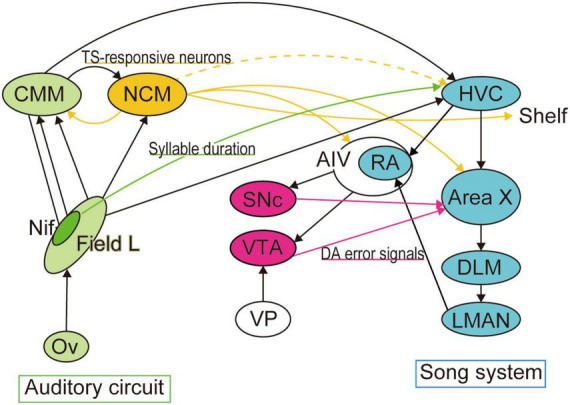
Neural circuits for template matching. Neural circuits necessary for song production and learning have been well identified as the song system (colored in cyan). Area X, the basal ganglia locus of the song system, is reported to receive dopaminergic error signals from VTA when auditory feedback is distorted. The premotor region of the song system, HVC, also receives sensory inputs from multiple auditory areas, including the telencephalic nucleus interface (NIf). Optogenetic activation of NIf projections to HVC encode the duration of song syllables. In contrast, accumulated studies have suggested that tutor memory forms in a higher auditory area, the caudal mesopallium (NCM), from which we found a transient projection to HVC during the song learning period.

HVC sits at the apex of the song system and constitutes the premotor area for singing behavior (Figure 2). Pioneering chronic multiunit recordings in HVC have shown firing activity when an adult male zebra finch sings (McCasland and Konishi, 1981). HVC comprises two types of projection neurons and interneurons and shows auditory responsiveness to playback of bird’s own songs (BOS) under anesthesia (Mooney, 2000). Other studies, including a detailed electrophysiological study with antidromic neuronal identification in awake singing birds, have revealed sequential sparse firing in a group of HVC neurons, which extends over the entire song duration (Hahnloser et al., 2002). Cooling HVC affects the temporal pattern of vocal behavior by slowing down the timing of the song (Long and Fee, 2008). Even in the absence of tutoring experiences, sequential activity in HVC can be observed, while pre-existing sequences become tightly associated with new own song after exposure to TS (Mackevicius et al., 2023). These series of studies have suggested that microcircuits within HVC regulate the generation of the song motor pattern. Perturbating the activity of the motor thalamic nucleus Uvaeformis (Uva), which projects to HVC, and imaging of Uva synaptic activity in HVC further support a model whereby thalamic input to “starter cells” in HVC drives sequential neuronal activity in the nucleus (Moll et al., 2023). Moreover, fractions of HVC neurons projecting to Area X (HVC_x_ neurons) have reported to work as “mirror neurons” (Prather et al., 2008; Fujimoto et al., 2011). Studies in swamp sparrows and Bengalese finches revealed that antidromically identified single HVC_x_ neuronal units exhibit neuronal firing both during singing and BOS playback, exactly the same time in the song phase, suggesting auditory-vocal information in single neurons. Notably, these studies were performed in adult zebra finches in which songs are “crystallized”. Contingent aversive stimulation in adult birds shifts the pitch of targeted syllables, but the pitch reverts to baseline when the perturbation is removed, suggesting that song templates are retained after song crystallization (Janata and Margoliash, 1999; Sober and Brainard, 2009). Is this template a TS auditory memory? Does the TS auditory template or the motor template (preactive or latent) guide song learning?

## Song motor plasticity driven by the anterior forebrain pathway (AFP)

Early studies on the lateral magnocellular nucleus of the anterior neostriatum (LMAN), the output region of the basal ganglia–thalamocortical loop of the song system, were implicated LMAN as the site associated with the tutor template. After LMAN lesions, juveniles loose motor plasticity which leads to poor song learning from TS (Bottjer et al., 1984; Scharff and Nottebohm, 1991). Juvenile LMAN neurons respond to playback of TS (Solis and Doupe, 1997, 1999). However, LMAN neurons do not respond to TS that is no longer similar to bird’s own song by re-learning from another tutor (Yazaki-Sugiyama and Mooney, 2004). Later studies have shown that LMAN contributes to acute song motor plasticity. LMAN activity is higher when zebra finches are singing undirected songs than direct songs, which are characterized by less variable acoustic features (Sossinka and Böhner, 1980; Jarvis et al., 1998; Hessler and Doupe, 1999). LMAN lesion reduces song variability in adults, suggesting defects in motor plasticity, similar to the previous finding in juveniles. Microsimulation of LMAN alters the song motor pattern (Kao et al., 2005). LMAN projects to a motor area, the robust nucleus of the arcopallium (RA), where neurons receive direct inputs from HVC. As described above, several studies have suggested that LMAN is responsible for error/error corrections (the output of a comparator). Still, important questions remain regarding the source of auditory feedback and the site of representation of the TS memory template. Additionally, whether the TS or motor template is compared to auditory feedback remains to be clarified.

## Auditory feedback to the song system

Recent studies using advanced techniques that allow manipulation of specific inputs to HVC have reported that optogenetical activation of the telencephalic nucleus interface (NIf) input at HVC synapses shapes the duration of syllables, while severing the NIf–HVC projection before, but not after, auditory learning from tutor disrupts song learning from tutor (Zhao et al., 2019). HVC rhythmic activity emerges in parallel with the emergence of new syllables during development (Okubo et al., 2015). Interestingly, *in vivo* intracellular recordings in awake zebra finches revealed that HVC neurons projecting to RA respond to TS in juveniles, but these responses are suppressed by local inhibitory circuits in adults (Vallentin et al., 2016).

Not only auditory feedback but dopaminergic signals have also been reported to shape song learning, as reported in reinforcement learning (Scharff and Nottebohm, 1991; Brainard and Doupe, 2000). The basal ganglia locus of the song system, Area X, receives dopaminergic inputs from the ventral tegmental area (VTA) (Person et al., 2008). VTA receives auditory inputs from the surrounding part of the arcopallium (ventral intermediate arcopallium [AIV]) (the “cup” adjacent to RA [RAcup]), suggesting a role in auditory feedback. Area X projects to the ventral pallidum (VP), which projects to VTA and the substantia nigra pars compacta (SNc) (Gale et al., 2008). VP receives inputs from AIV and sends projections to HVC and RA (Li and Sakaguchi, 1997). This architecture collectively demonstrates that the basal ganglia–thalamocortical pathway forms a loop with dopaminergic inputs. Inactivating VTA neurons in Bengalese finches (*Lonchura striata var. domestica*) disrupts the ability of the birds to shift pitches of songs to avoid aversive stimulation (Hoffmann et al., 2016). Electrophysiological recordings from VTA neurons have revealed a role in computing performance error signals upon distorted auditory feedback (Gadagkar et al., 2016) and natural fluctuations in performance of VTA neurons projecting to Area X (Duffy et al., 2022). Dopaminergic signals in Area X are depend on performance, and diminishing during courtship (Roeser et al., 2023). To compute error-based reinforcement signals, neurons require inputs from both auditory feedback of own vocalization and a template (target motor pattern). A question remains unresolved: is this template a memory of TS or a motor template? The studies described here were performed in adult birds, which raises the issue of whether the template matching systems during juvenile song learning and adult song maintenance overlap.

## Auditory memory in auditory forebrain: song memory and song discrimination

As described in the previous sections, auditory guiding/feedback signals to HVC appear to instruct song learning, while performance error is computed in the VTA–Area X circuit. Except for the information on syllable length from NIf to HVC, the type of information that arrives at HVC or VTA for template matching and its source remains largely unknown. In addition to research on the song system, more recent studies have shown that TS memories are stored in brain regions within the auditory pathways, especially in higher auditory areas. The expression level of the immediate-early gene, *ZENK*, a molecular marker for neuronal activity, in the zebra finch higher auditory area, the caudomedial nidopallium (NCM), is higher in birds exposed to TS playback than in those exposed to unfamiliar zebra finch songs (Gobes et al., 2010). In both sexes, NCM has been suggested to be the site of memory storage of auditory experiences, not exclusively for song learning. NCM lesions after conditioning with song stimulation diminishes song discrimination ability in adult males (Gobes and Bolhuis, 2007; Canopoli et al., 2014; Yu et al., 2023), while lesions in adult females disrupt song preference to experienced songs (Tomaszycki and Blaine, 2014).

A series of studies have suggested the involvement of NCM in song learning as a TS memory brain region. ZENK expression levels upon TS exposure positively correlate with the amount of song learning from a tutor (Bolhuis et al., 2000, 2001; Terpstra et al., 2004). Pharmacological blockade of a signaling pathway in NCM prevents juveniles to learn from TS (London and Clayton, 2008). An electrophysiological study revealed distinct habituation rates in NCM auditory responses upon repeated exposure to TS and unfamiliar song (Phan et al., 2006). We have reported that a small subset of juvenile NCM neurons show almost exclusive auditory responsiveness to a learned TS (Yanagihara and Yazaki-Sugiyama, 2016; Katic et al., 2022). In contrast to the other brain loci in the song system, nearly all neurons are selective to the bird’s own song (Doupe and Konishi, 1991; Solis and Doupe, 1997; Janata and Margoliash, 1999). In NCM, electrophysiological experiments revealed two types of neurons distinct in their spiking shapes and firing rates (Schneider and Woolley, 2013; Yanagihara and Yazaki-Sugiyama, 2016), including inhibitory neurons (Spool et al., 2021). Only a subset (∼15%) of broader spiking NCM neurons acquire selectivity to TS soon after (∼1 h) listening to tutor singing (Katic et al., 2022). This timeline parallels a previous finding, in that TS memory forms by hearing only a few renditions of songs. The responsiveness of these neurons is exclusive to TS and not even to birds’ own songs, suggesting that they comprise the neuronal substrates of auditory memory.

Despite cumulative studies that has implicated the zebra finch NCM in memory formation, neither a direct anatomical connection between NCM and the song system (Vates et al., 1996) nor instructive NCM neuronal activity during juvenile singing has been elucidated. NCM has reciprocal connections with the caudomedial mesopallium (CMM) which projects to HVC (Vates et al., 1996). While AFP in the song system receives dopaminergic reinforcement signals for song learning as discussed in the previous paragraph, NCM receives inputs from the noradrenergic locus coeruleus (LC), the brain region that controls attention and arousal states and noradrenergic release (Velho et al., 2012). LC to NCM inputs are suggested to send social information for song learning (Katic et al., 2022). These connectivity patterns collectively show that the song system and auditory pathway are integrated with a neuromodulatory system (Figure 2). Using viral technology to manipulate gene expression in target neurons and whole-brain axonal tracing with tissue clearing, we recently reported a transient projection to HVC from the subset of NCM neurons responsive to TS playback. The TS-responsive NCM neurons project to HVC, HVC-shelf, AIV, CMM, and Area X in juveniles, but the HVC projection disappears in adults. Inducing cell death in these NCM TS-responsive neurons by targeting the expression of CaCaspase disrupts song learning in juveniles but not in adults (Louder et al., 2024). While these results do not specify which of the projections from NCM are necessary for song learning, the NCM–HVC temporal connections comprise a candidate circuit for auditory TS memory-guided sensorimotor learning; moreover, dynamic rewiring of the interareal neural circuit may regulate the developmental time course of song learning. This raises the question of the fate of TS auditory memories over the developmental period.

## Discussion

Whether a brain area is related to the process of distinguishing individual songs or in memory formation and storing cannot be decidedly determined in lesion experiments. Rather than disrupting auditory memories, the lesion experiments described above might have interrupted song discrimination. Sensory memories are thought to last long, perhaps permanently. In some bird species, sensory and sensorimotor learning is separated by 2–3 months, suggesting (at a minimum) month-long auditory memories. In zebra finches that learned two songs sequentially from two distinct tutors, the level of Zenk expression upon exposure to TS correlates with the amount of song learning from either tutor (Olson et al., 2016), suggesting that NCM is the substrate for TS memory encoding in adults, while neuronal substrates for TS memory in adults have yet to be identified. Auditory memories of TS are necessary for song learning, but storage may occur in different brain regions in juveniles and adults, specifically before and after song learning.

Similarly, while auditory learning can be extended if TS are not provided (e.g., in isolation), the song crystallizes at a specific developmental time regardless of the level of learning. Even in the absence of TS experiences, juvenile birds start to sing at a specific time point during development. These observations suggest that the time course of sensory and sensorimotor learning are independently regulated but well-coordinated. Forming a memory during early auditory learning is necessary and TS memories are thought to guide sensorimotor learning. The higher auditory area is a strong, but likely not exclusive, candidate as the locus of TS memory encoding. Moreover, different brain regions may be responsible for the generation of auditory memory to guide sensorimotor learning during development and song recognition in the latter stage. Detailed dissections of neural circuits over the entire song learning period and manipulation of specific circuits during song learning are expected to enrich knowledge on the song template, TS template, or own song motor template systems, and provide insights into the relevant spatial and temporal characteristics in the brain. Furthermore, such studies likely have implications for bilingualism in humans. Early experiences of adults in non-native language settings have positive influences on auditory discrimination ability of bowling or intonations even when speaking ability of the second language is limited. Retention of connections that guide the flow from auditory memories to the motor area may be a key factor for re-opening re-learning or new song learning in adults. Hearing is one thing, doing (mimicking) might be another. The long-existing song template theory should be explored in more depth to thoroughly understand its concepts.

## Author contributions

YY-S: Conceptualization, Writing–original draft, Writing–review and editing.
